# Treatment of Anasarca by the Subcutaneous Injection of Antistreptococcus Serum (Marmorek)

**Published:** 1897-02

**Authors:** Harold Sorby

**Affiliations:** Chicago, Ill.


### TREATMENT OF ANASARCA BY THE SUBCUTANEOUS INJECTION OF ANTISTREPTOCOCCUS SERUM (MARMOREK).

By Harold Sorby,
CHICAGO, ILL.

A recent communication from the Pasteur Institute, Paris, announces the important fact that anasarca in horses is due to a variety of the microbe known as streptococcus, and that the most rational as well as the most successful treatment for that condition is the hypodermic injection of the antistreptococcus serum (Marmorek). The communication referred to says, further, that by the intelligent employment of this remedy the microbes are quickly destroyed, resolution established, and all the symptoms materially modified. A few days only are required to bring about a noticeable improvement, such as progressive diminution of the swellings, disappearance of petechiæ, and lowering of the temperature. The last named, however, is not of vital importance, as an elevation of temperature may be due to some concurrent infection. The specific cause being removed, the after-treatment is very much simplified, judicious feeding and general tonics usually fulfilling all requirements.

The serum mentioned has already been extensively used in the human subject in the treatment of erysipelas, puerperal septicæmia, etc., and with universal success. If it shall now prove to be a specific for equine anasarca as well, it must rank as one of the most valuable discoveries in sero-therapy.

